# Fisetin glycosides synthesized by cyclodextrin glycosyltransferase from *Paenibacillus* sp. RB01: characterization, molecular docking, and antioxidant activity

**DOI:** 10.7717/peerj.13467

**Published:** 2022-05-24

**Authors:** Nattawadee Lorthongpanich, Panupong Mahalapbutr, Thanyada Rungrotmongkol, Thanapon Charoenwongpaiboon, Manchumas Hengsakul Prousoontorn

**Affiliations:** 1 Department of Biochemistry, Faculty of Science, Chulalongkorn University, Bangkok, Thailand; 2 Department of Biochemistry, and Center for Translational Medicine, Faculty of Medicine, Khon Kaen University, Khon Kaen, Thailand; 3 Structural and Computational Biology Research Unit, Department of Biochemistry, Faculty of Science, Chulalongkorn University, Bangkok, Thailand; 4 Department of Chemistry, Faculty of Science, Silpakorn University, Nakhon Pathom, Thailand

**Keywords:** Cyclodextrin glycosyltransferase, Fisetin, Antioxidant, Transglycosylation

## Abstract

Fisetin is a flavonoid that exhibits high antioxidant activity and is widely employed in the pharmacological industries. However, the application of fisetin is limited due to its low water solubility. In this study, glycoside derivatives of fisetin were synthesized by an enzymatic reaction using cyclodextrin glycosyltransferase (CGTase) from *Paenibacillus* sp. RB01 in order to improve the water solubility of fisetin. Under optimal conditions, CGTase was able to convert more than 400 mg/L of fisetin to its glycoside derivatives, which is significantly higher than the previous biosynthesis using engineered *E. coli*. Product characterization by HPLC and LC-MS/MS revealed that the transglycosylated products consisted of at least five fisetin glycoside derivatives, including fisetin mono-, di- and triglucosides, as well as their isomers. Enzymatic analysis by glucoamylase and α-glucosidase showed that these fisetin glycosides were formed by α-1,4-glycosidic linkages. Molecular docking demonstrated that there are two possible binding modes of fisetin in the enzyme active site containing CGTase-glysosyl intermediate, in which O7 and O4’ atoms of fisetin positioned close to the C1 of glycoside donor, corresponding to the isomers of the obtained fisetin monoglucosides. In addition, the water solubility and the antioxidant activity of the fisetin monoglucosides were tested. It was found that their water solubility was increased at least 800 times when compared to that of their parent molecule while still maintaining the antioxidant activity. This study revealed the potential application of CGTase to improve the solubility of flavonoids.

## Introduction

Fisetin (3,3′,4’,7-tetrahydroxyflavone) is a flavonoid found in many plants such as strawberries, apples, persimmons, onions, and cucumbers. It exhibits several biological activities, including antioxidant, anti-ageing, anticancer, antiviral, antiallergic and neuroprotective functions (Khan et al., 2013; Lorthongpanich et al., 2021; Ruela de Sousa et al., 2007). Nevertheless, the use of fisetin is limited due to its low bioavailability and solubility in water. The solubility and bioavailability of fisetin have been improved by several methods. For instance, liposomal encapsulation using the amphiphilic surface of phospholipids (Seguin et al., 2013), complexation with the cationic dimer of cyclosophoraose (Jeong et al., 2013), and complexation with nanoparticulated inulin (Charoenwongpaiboon et al., 2019).

Enzymatic transglycosylation is a method that has been employed to improve the water solubility of many bioactive compounds, including flavonoids and polyphenols. The enzymatic reaction has an advantage over the chemical reaction because of its specificity and mild reaction condition. The glycosyl units are transferred from the glycosyl donor to an acceptor (a compound containing hydroxyl group) using the activity of glycosyltransferase such as dextransucrase (Kim et al., 2012; Klingel et al., 2019), amylosucrase (Jang et al., 2018), levansucrase (Núñez-López et al., 2019) and cyclodextrin glycosyltransferase (Aramsangtienchai et al., 2011; Torres et al., 2011). Since the enzyme is specific towards transglycosylation position of an acceptor molecule, the use of different glycosyltransferase enzymes might lead to a wide variety of glycosylated products with different functionalities.

Cyclodextrin glycosyltransferase (CGTase, E.C. 2.4.1.19) is an enzyme that can synthesize cyclodextrins (CDs) and oligosaccharides of α-1,4-linked glucose from starch *via* cyclization and disproportionation reactions, respectively (Leemhuis, Kelly & Dijkhuizen, 2010; Qi & Zimmermann, 2005). CGTase also catalyzes the coupling reaction and hydrolysis (Svensson, Ulvenlund & Adlercreutz, 2009). The coupling reaction is a type of transglycosylation when CDs are used as glycosyl donors. CDs can form an inclusion complex with various compounds and are widely used as solubilizers and stabilizers, which makes CDs have numerous applications in the food, cosmetics, and pharmaceutical industries (Mahalapbutr et al., 2021; Mahalapbutr et al., 2019b). The formation of the fisetin/cyclodextrin inclusion complex was also reported in order to improve the property of fisetin (Zhang et al., 2015). CGTase can be found in various bacterial strains such as *Bacillus* (Arce-Vázquez et al., 2016; de Araújo Coelho et al., 2016; Tao et al., 2019; van der Veen et al., 2000), *Pseudomonas* (Jiang et al., 2018), and *Thermoanaerobacterium* (Leemhuis, Dijkstra & Dijkhuizen, 2003). CGTase was used to synthesize various flavonoid glycosides using various flavonoid acceptors such as hesperidin (Kometani et al., 1994), hesperetin (González-Alfonso et al., 2018), naringin (Kometani et al., 1996), epicatechin (Aramsangtienchai et al., 2011) and resveratrol (Torres et al., 2011). However, to the best of our knowledge, the synthesis of fisetin glucosides using CGTase has not yet been studied. In previous study, our research group has isolated, cloned, and characterized the CGTase from *Paenibacillus* sp. RB01, a microorganism derived from hot spring soil (Charoensakdi et al., 2007; Yenpetch et al., 2011a; Yenpetch et al., 2011b). The RB01 CGTase was used to synthesize alkyl glycosides using alcohols as acceptors (Chotipanang, Bhunthumnavin & Prousoontorn, 2011). These results revealed that this CGTase is stable in a co-organic solvent and at high temperature. Therefore, in this study, fisetin glycosides were synthesized by the transglycosylation reaction of *Paenibacillus* sp. RB01 CGTase using β-cyclodextrin (β-CD) as a glucosyl donor. The reaction was performed in a co-organic solvent to improve the solubility of fisetin. The products were purified, and their structures were analyzed by enzymatic hydrolysis and LC-MS/MS. The water solubility and antioxidant activity of the glycoside products were also measured and compared to those of the parent fisetin. In consequence, data reported in this paper could reveal the potential application of CGTase for commercial developments of novel flavonoid glycosides.

## Materials and Methods

### Material and chemicals

Fisetin, β-cyclodextrin, glucoamylase (*Aspergillus niger*), α-glucosidase (*Saccharomyces cerevisiae*) and 2,2-diphenyl-1-picrylhydrazyl (DPPH) were purchased from Sigma-Aldrich (Sigma-Aldrich, St. Louis, MO, USA). Acetonitrile (HPLC grade) was from RCI Labscan (RCI Labscan, Bangkok, Thailand). All other chemicals were of analytical grade.

### Enzyme production and purification

*Paenibacillus* sp. RB01 isolated from hot spring soil from Ratchaburi province, Thailand, was used for CGTase production (Charoensakdi et al., 2007). Bacteria was streaked on solid medium I (0.5% (w/v) beef extract, 1.0% (w/v) peptone, 0.2% (w/v) NaCl, 0.2% (w/v) yeast extract and 1.0% (w/v) soluble starch) and incubated at 37 °C for 18 h. A colony was inoculated into medium I broth and shaken at 37 °C, 250 rpm in orbital shaker until OD_660_ reached 0.3–0.5. The obtained starter inoculum of 1.0% (v/v) was transferred into Horikoshi’s medium (1.0% (w/v) soluble starch, 0.5% (w/v) yeast extract, 0.1% (w/v) K_2_HPO_4_, 0.02% (w/v) MgSO_4_, 0.5% (w/v) peptone and 0.75% (w/v) Na_2_CO_3_) and cultivated at 37 °C, 250 rpm in shaking incubator for 72 h. The cells were separated by centrifugation at 8,000×g at 4 °C for 15 min. Supernatant containing the secreted extracellular CGTase was collected and kept at 4 °C until further used.

The crude extract of CGTase was partially purified by the starch adsorption method (Martins & Hatti-Kaul, 2003). In brief, the commercial-grade corn starch (Knorr™; Unilever Thai Holding Co, Ltd., Bangkok, Thailand) was dried at 121 °C for 30 min before slowly being added into crude CGTase solution to reach a final concentration of 5% (w/v). The starch suspension was stirred at 4 °C overnight. The starch was harvested by centrifugation at 8,000×g, at 4 °C for 30 min. The obtained starch cake was washed with cool TB1 buffer (10 mM Tris-HCl (pH 8.5) containing 10 mM CaCl_2_) until the solution was clear. CGTase was eluted from starch using TB2 buffer (0.2 M maltose in TB1 buffer). The eluent was collected by centrifugation at 8,000×g, at 4 °C for 30 min. The supernatant was dialyzed against 50 mM phosphate buffer (pH 6.0) at 4 °C.

### Enzyme activity assay

The CGTase activity was measured by starch hydrolytic activity assay (dextrinizing activity) using iodine (Rimphanitchayakit, Tonozuka & Sakano, 2005). In brief, 50 µL of enzyme sample was incubated with 150 µL of 0.2% (w/v) soluble starch in 0.2 M phosphate buffer (pH 6.0) at 40 °C for 10 min. The reaction was then terminated by the addition of 2 mL of 0.2 M HCl, followed by 250 µL of iodine reagent (0.02% (w/v) I_2_ in 0.2% (w/v) KI). The reaction was adjusted to a final volume of 5 mL with distilled water and the absorbance at 600 nm was read by a spectrophotometer. One unit of the enzyme was defined as the amount of enzyme which reduced 10% of the blue color (A600) of starch-iodine complex per minute.

### Effect of cosolvent on CGTase stability

The effect of cosolvent on CGTase stability was studied using acetone and dimethyl sulfoxide (DMSO). Nine hundred units per milliliter of CGTase was incubated in 50 mM phosphate buffer (pH 6.0) containing various concentrations of acetone or DMSO (0–50% (v/v)) at 40 °C for 24 h. The remaining activity of CGTase was measured as the method described above. Experiments were performed in triplicate. Error bars represent standard deviation (SD).

### Fisetin glycoside synthesis

Fisetin glycosides were synthesized using β-CD as a glycosyl donor. Fisetin (0.25 % (w/v)) and β-CD (1.0% (w/v)) were incubated with CGTase (200 U/mL) in 50 mM phosphate buffer (pH 6.0) containing different concentration of DMSO (10–50% (v/v)) at 40 °C for 24 h. The reaction was terminated by boiling for 10 min and the glycoside products were analyzed by thin-layer chromatography (TLC) and high-performance liquid chromatography (HPLC).

### TLC, HPLC-RID and LC-MS/MS analysis

The products were preliminarily analyzed by TLC using silica gel 60 F254 aluminum sheets (Merck, Darmstadt, Germany). Samples were loaded onto a TLC plate and were separated with ethyl acetate: methanol: water: toluene (10: 1.5: 1.3: 0.2 by volume). The product spots were visualized by UV lamp (360 nm) and by spraying with orcinol (27 mL of ethanol, 10 mL of conc. H_2_SO_4_, 8 mL of water, and 0.1 g of orcinol) and heating at 120 °C for 10 min)). The semi-quantitation of fisetin glycosides can be performed by measuring the spot intensities using Quantity One software (Bio-Rad, Hercules, CA, USA).

HPLC equipped with a photodiode array detector (PDA) (Shimadzu, Kyoto, Japan) was used to quantitate the amount of fisetin and their glycoside derivatives. Fisetin glycoside products were separated by C18 column (C18P 4E, 4.6 × 250 mm, SHODEX) at 40 °C using the gradient between SolA (acetonitrile) and SolB (0.1% (v/v) formic acid) at a flow rate of 1 mL/min for 25 min. The gradient program was described as follow: 20% of SolA (0–4 min), 40% of SolA (4–8 min), 100% of SolA (8–14 min), 50% of SolA (14–17 min) and 20% of SolA (17–25 min). Fisetin and its glycosides were detected at 350 nm. The amount of the glycoside products was calculated based on the peak area using fisetin as an external standard.

The structures of fisetin glycosides were examined by LC-MS/MS (TSQ Endura™ triple Quadrupole Mass Spectrometer; ThermoFisher SCIENTIFIC, Waltham, MA, USA). The compounds were separated using the same system as described above and ionized by electrospray ionization on negative-ion mode. The structure of fisetin glycoside products were evaluated from the fragmentation pattern of MS/MS spectrum.

### Purification of fisetin glycosides

The obtained fisetin glycosides were purified by solvent extraction and HPLC. First, the reaction mixture was extracted twice with an equal volume of butyl acetate. The aqueous phase was collected, pooled, and concentrated by lyophilization. The derived fisetin glycoside extract was further purified by HPLC on a C18 column equipped with a fraction collector. Products were separated by using a gradient of 0.1% (v/v) of formic acid and acetonitrile as described above.

### Evaluation of the transglycosylation products using enzymatic hydrolysis

Transglycosylation products were treated with a final activity of 60 U/mL of glucoamylase in 50 mM acetate buffer (pH 6.0) at 40 °C for 4 h. The reaction was terminated by boiling for 10 min and drying. After that, the samples were incubated with 60 U/mL of α-glucosidase at a final concentration in 50 mM acetate buffer (pH 6.0) at 40 °C for 16 h. The reaction was stopped by boiling for 5 min. The derived hydrolytic reactions were analyzed by HPLC.

### Water solubility study

The molar concentration of dissolved fisetin monoglucosides was determined from fisetin concentration after acidic hydrolysis. In brief, the purified fisetin glucoside products were dissolved in 50 µL of deionized water at 25 °C. The undissolved fraction was separated from the solution by centrifugation at 10,000×g for 10 min. The glucoside samples in the supernatant were hydrolyzed using 1.2 M HCl in 50% (v/v) methanol at 100 °C for 10 min. The reactions were then neutralized with 2 M Tris base solution in 50% (v/v) methanol. The amount of fisetin released was then analyzed by HPLC using fisetin as an external standard.

### Molecular docking

The homology model of *Paenibacillus* sp. RB01 CGTase was generated by the SWISS-MODEL server (Schwede et al., 2003) using the crystal structure of the complex between a covalent intermediate and *Bacillus circulans* strain 251 CGTase (PDB ID: 1CXL) as a template. Then, the structural superimposition between the model and the crystal structure of *Bacillus circulans* strain 251 CGTase was conducted to obtain the atomic coordinate of glucosyl moiety. The initial structure of fisetin was created and optimized using Gaussian 09 program as per the standard protocols (Frisch et al., 2016; Mahalapbutr et al., 2019a; Mahalapbutr et al., 2019b). Subsequently, the protein-ligand complex was generated using the FlexX docking program (Kramer, Rarey & Lengauer, 1999) (500 docking runs and spherical radius of 15 Å).

### Assay for antioxidant activity

The antioxidant activities were determined using DPPH assay (Pyrzynska & Pękal, 2013). One hundred microliters of DPPH solution (0.015 mg/mL) were added into 96 well-plates. After that, the various concentration of sample solution, namely fisetin, purified glycoside product, and ascorbic acid, were added and mixed with the former DPPH solution to reach the final volume of 250 µL. The plate was kept in the dark for 30 min before being subjected to the absorbance measurement at 520 nm using a microplate reader (SYNERGY). Blank for the reactions was the solvent that dissolved the samples (methanol or water). The radical scavenging activities (RSA) of each sample were calculated according to the formula as follow:

$\rm{\%}{RSA} = {{[{(AB-AA)/AB}]} \times 100}$

where %RSA is percentage inhibition, AB and AA are the absorbance values of the blank sample and the sample, respectively. The graph of %RSA *versus* sample concentrations was plotted. Linear regression analysis was carried out in order to calculate the effective concentration of the sample required to scavenge DPPH radical by 50% (IC50). Experiments were performed in triplicate. Error bars represent standard deviation (SD).

## Results and Discussion

### Production and partial purification of CGTase from *Paenibacillus* sp. RB01

CGTase from *Paenibacillus* sp. RB01 was successfully expressed in Horikoshi’s medium with a specific dextrinizing activity of 146 U/mg protein. The crude CGTase can merely be purified by a single step using the starch adsorption method because it contains a high-affinity starch binding motif. The result of the purification is summarized in Table S1. The enzyme was purified 34-fold with a yield of 31% of the activity in the crude enzyme. The specific activity of the purified enzyme increased to 5,030 U/mg. SDS-PAGE showed the intense band at 66 kDa, which corresponded to the molecular weight of CGTase (Fig. S1). As a result, it demonstrated that the starch adsorption method is straightforward and effective.

### Effect of cosolvent on CGTase stability and fisetin glycoside synthesis

**Figure 1:**
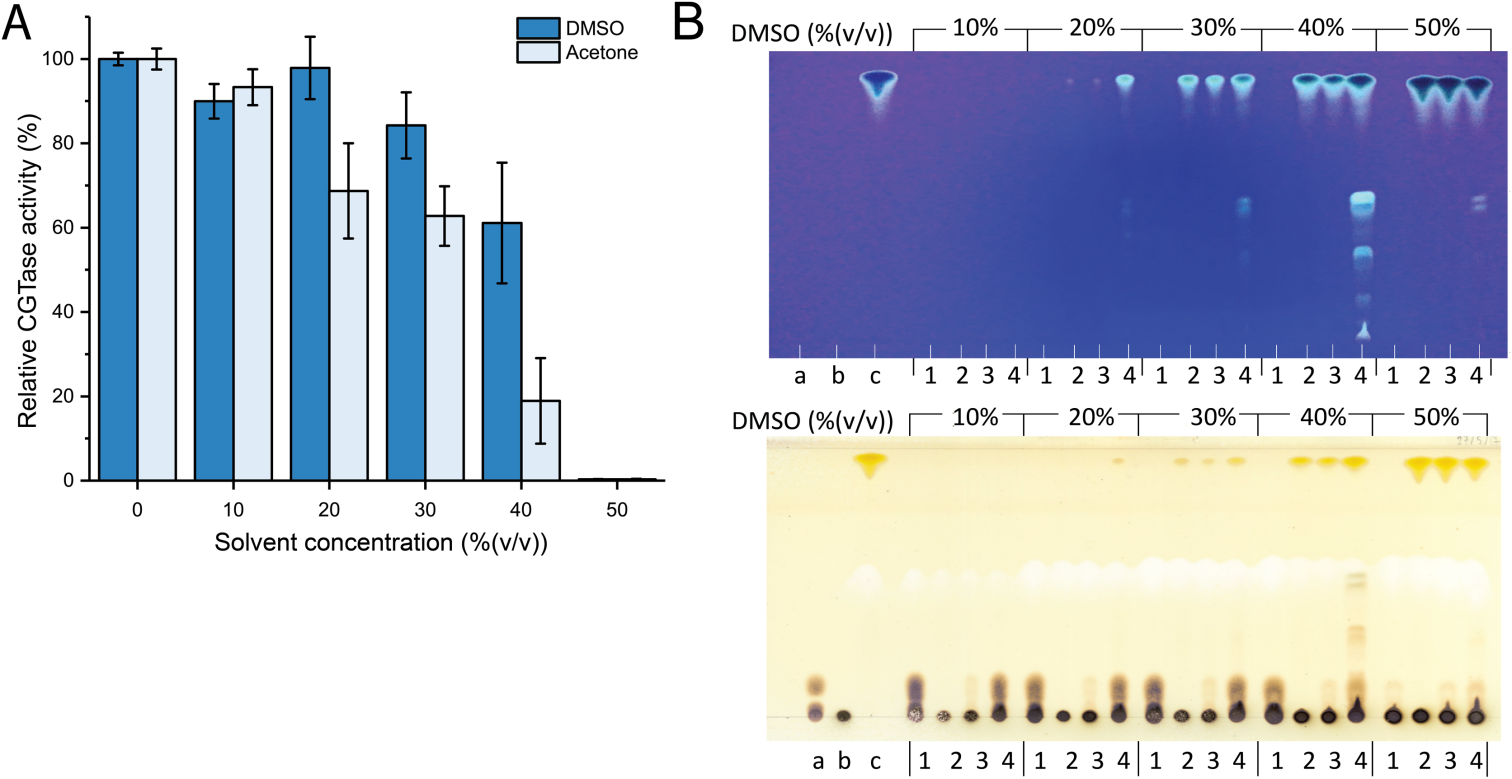
The effect of organic solvents on stability and transglycosylation of CGTase. (A) The relative activity of CGTase after incubation at 40 °C for 24 h. The activity of the enzyme was measured by starch hydrolysis assay. (B) TLC analysis of transglycosylation products of CGTase. The reactions were performed using 1% (w/v) β-CD in phosphate buffer, pH 6.0 containing different concentration of DMSO (10–50% (v/v)) at 40 °C for 24 h. Lane a, G1–G3 oligosaccharide standards; lane b, β-cyclodextrin; lane c, fisetin; lane 1, the reaction without fisetin; lane 2 the reaction without enzyme; lane 3, the reaction at 0-h incubation; lane 4, the reaction at 24-h incubation. Spots were detected by UV lamp (top) and spraying with orcinol (bottom).

Since fisetin has low solubility in water, the synthesis of fisetin glycosides in an aqueous solution might be inefficient. To improve the solubility of fisetin, the reaction was performed in the cosolvent system. DMSO and acetone were candidates because they do not contain any hydroxyl groups and thus, cannot act as glycosyl acceptors. However, the use of organic solvents usually causes the denaturation or aggregation of the enzyme, resulting in the reduction of enzyme activity. Therefore, the stability of CGTase in given organic solvents must be explored. The results showed that CGTase retained higher activity in DMSO than that in acetone (Fig. 1A). The enzyme retained more than 80% of initial activity after incubation in 30% (v/v) DMSO and retained more than 50% of initial activity after incubation in 40% (v/v) DMSO, suggesting that DMSO was more suitable for CGTase to transglycosylate fisetin.

Attempts were then made to synthesize the fisetin glycoside using different concentrations of DMSO (10–50% (v/v)). The reactions were performed using CGTase and fisetin at a final concentration of 200 U/mL and 0.25% (w/v), respectively. The products were then analyzed by TLC. As a result, at least five expected products were detected. The fluorescent bands on the TLC indicated that the compounds contain a conjugated system, which is undeniably to be fisetin, while the bands detected by orcinol sprayed indicated that these compounds contained glycoside moieties (Fig. 1B). The intensity of the expected product bands increased with DMSO concentration and reached its maximum at 40% (v/v) of DMSO (Fig. 1B). This result can be explained by the fact that at higher DMSO concentration, the solubility of fisetin was increased, resulting in a higher concentration of products obtained. However, the intensity of product bands dramatically decreased when 50% (v/v) of DMSO. This might be a result of the denaturation of the enzyme by the high concentration of organic solvent, causing the enzyme inactivation. This result correlated to the finding above that CGTase almost lost all their activity at 50% (v/v) DMSO. According to previous research, CGTase could retain high activity in cosolvent system (Blackwood & Bucke, 2000; Doukyu, Kuwahara & Aono, 2003). CGTase from *Paenibacillus illinoisensis* ST-12 K retained 78–100% of initial activity in various water-miscible cosolvent system such as methanol, ethanol and 2-propanol (Doukyu, Kuwahara & Aono, 2003), while the activity of many glycosyltransferases largely decreases at the high concentration of organic solvents. For example, 6-O-α-rhamnosyl-βglucosidase from *Acremonium* sp. retained only 57% of initial activity in 30% (v/v) DMSO (Mazzaferro et al., 2019), β-fructosidase from *Arthrobacter nicotianae* XM6 retained 25% of catalytic activity in 20% (v/v) DMSO (Wu et al., 2013), and maltogenic amylase from *Parageobacillus galactosidasius* DSM 18751 exhibited less than 50% of initial activity in 30% (v/v) DMSO (Wu et al., 2021). Commonly, cosolvent was used to improve the solubility of flavonoids for enhancing the transglycosylation yield (Ji et al., 2020). Hence, organic solvent tolerant CGTase should have a high potential for synthesis of flavonoid glycosides.

The effects of the reaction condition, including fisetin, β-CD, and enzyme concentration, were investigated. It was found that substrate concentrations (fisetin and β-CD) strongly affected the yield of fisetin glycosides synthesized by CGTase (Fig. S7). The higher the β-CD concentration was used, the higher amount of fisetin glycosides was produced. This finding corresponded to a previous study in which the transglycosylation reaction was mostly dependent on initial glycosyl donor concentration (Kometani et al., 1994; Moreno et al., 2010). However, the increase of fisetin concentration showed a different effect. Fisetin conversion was optimum at 0.25% (w/v) fisetin and decreased when a higher concentration of fisetin was used. This might be the limitation of fisetin solubility in cosolvent, or inhibitory effect of fisetin on CGTase activity. Meanwhile, fisetin conversion was seen to decrease when more than 200 U/mL of CGTase was applied. A high concentration of enzyme may reduce the enzyme solubility in cosolvent and cause the formation of enzyme aggregate (Li et al., 2007; Treuheit, Kosky & Brems, 2002; Ye et al., 2014). Under optimum condition (0.25% (w/v) fisetin, 1% (w/v) β-CD, 200 U/mL CGTase and 24 h incubation time), 17% (425 mg/L) of supplemented fisetin was converted to fisetin glycosides. Although CGTase synthesized relatively low yield of fisetin glycosides, enzymatic glycosylation of fisetin still had a benefit over bacterial conversion. Enzymatic glycosylation is easy to operate, and the obtained products are simple to purify. In comparison with the previous study, the metabolically engineered *Escherichia coli* that overexpresses thymidine diphosphate (dTDP)-d-glucose synthase (tgs), dTDP-d-glucose 4,6-dehydratase (dh), and a sugar aminotransferase (wecE) converted only 4.29 mg/L of fisetin within 48 h (0.09 mg L^−1^ h^−1^) (Pandey et al., 2016). Another metabolically engineered *E. coli* assembling multiple genes of a nucleotide diphosphate (NDP)-sugar biosynthetic pathway could converted 95% of fisetin to fisetin glycosides within 48 h (Parajuli et al., 2015). Although very high conversion yield was obtained, this *E. coli* catalyzed the reaction at slower rate (6.25 mg L^−1^ h^−1^) and utilized lower total fisetin concentration (285 mg/L). The higher conversion rate (17.7 mg L^−1^ h^−1^) of CGTase and the obtained fisetin glycoside (1.5 mM; equal to 425 mg/L fisetin) makes this biocatalyst attractive for the synthesis of other flavonoid glycosides. Moreover, CGTase uses cheap substrates, such as starch or cyclodextrin, as glycosyl donors. It is reasonable to use this enzyme for scaling up the production of fisetin glycosides.

**Figure 2:**
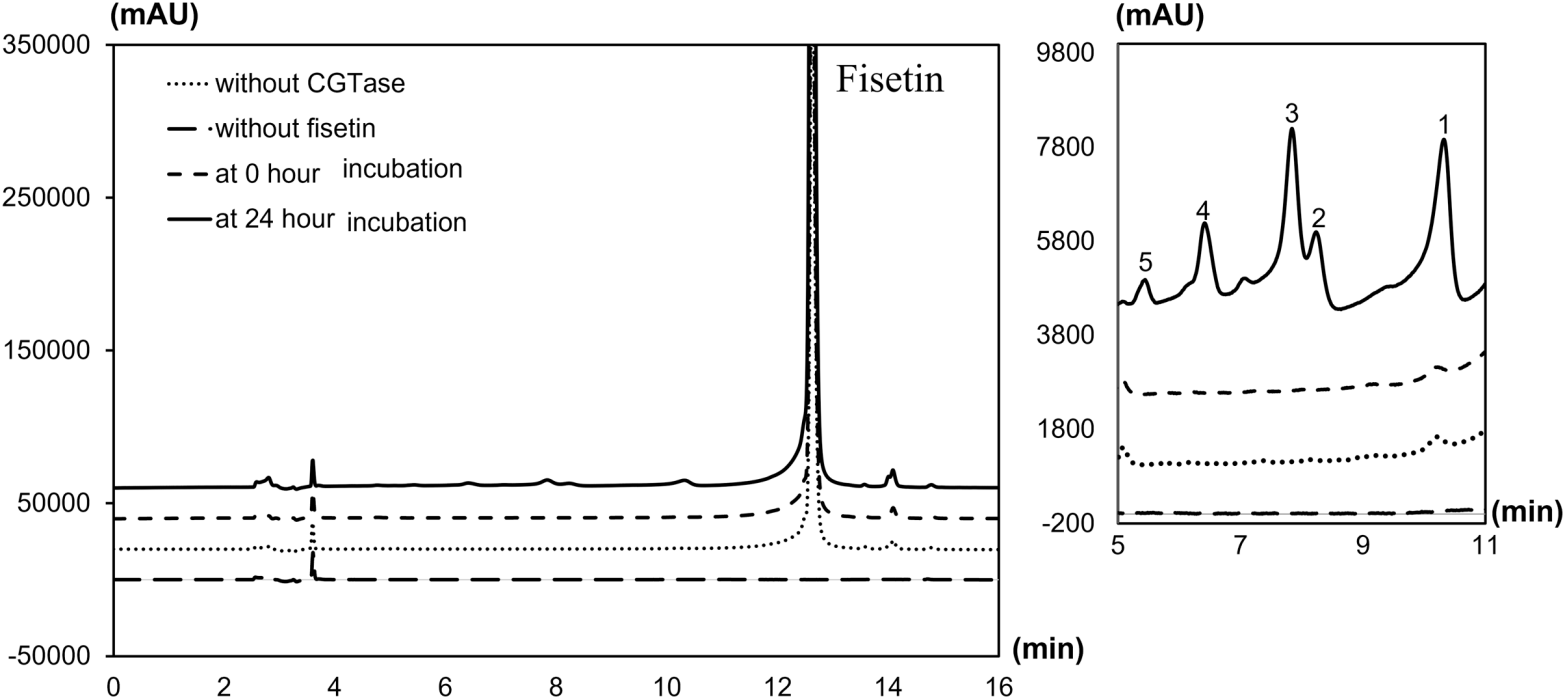
HPLC chromatogram of fisetin glycosides synthesized from fisetin and β-CD by CGTase. Number on the top of each peak referred to each glycoside product that was subsequently subjected to ESI-MS analysis.

The degree of glycosylation to fisetin by CGTase was also determined by HPLC (Fig. 2). HPLC chromatogram also showed a series of transglycosylated products of fisetin which could be synthesized by this enzymatic system. Each product peak was collected by HPLC equipped with a fraction collector and further analyzed by MS in the negative ionization mode. Mass spectroscopy analysis showed that product 1 (RT 10.3 min) and product 3 (RT 7.8 min) had a total mass of m/z = 447.08, which corresponded to the molecular weight of fisetin mono-glucoside. Meanwhile, the total mass of product 2 (RT 8.2 min) and product 5 (RT 5.5 min) were correlated to the molecular weight of fisetin di-glucoside (m/z = 609.14), and product 4 (RT 6.4 min) was fisetin triglucoside (m/z = 771.19) (Figs. S2–S6). These results indicated that CGTase could transfer the glucose to at least two different positions of fisetin. The two fisetin glycosides with similar molecular mass were detected, but they were eluted from HPLC at different retention time. This result suggested that they may have different polarities, which was dependent on glycosylation position.

### Structural determination of derived fisetin glycosides

**Figure 3:**
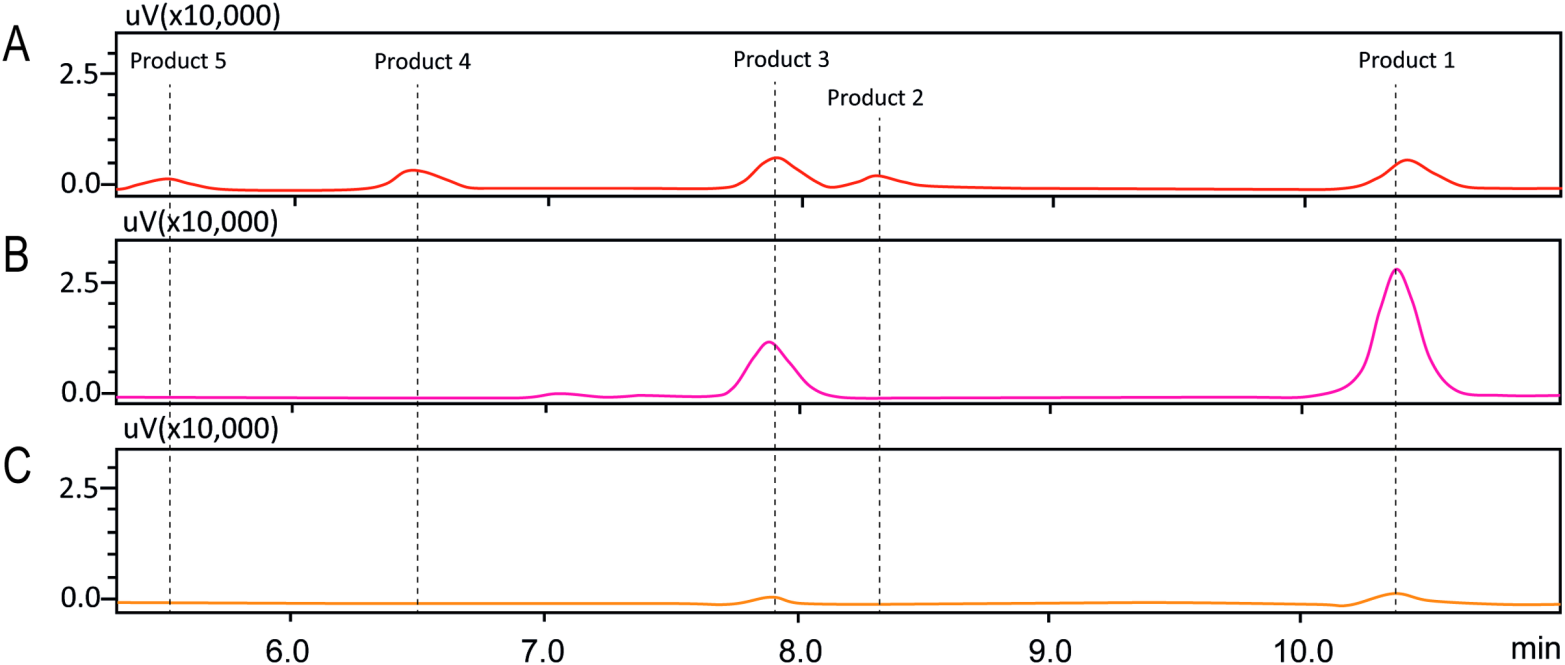
HPLC chromatograms of enzymatic analysis of obtained fisetin glycosides. (A) Initial translgycosylated products; (B) products hydrolyzed by glucoamylase; and (C) products hydrolyzed by glucoamylase and α-glucosidase.

In order to investigate the structure of obtained fisetin glycosides, the samples were first subjected to enzymatic hydrolysis by glycosyl hydrolases. Glucoamylase and α-glucosidase were used to identify the α-linkage between glucose-glucose and fisetin-glucose, respectively (Fig. 3) (Aramsangtienchai et al., 2011; Chiba, 1997). When the product mixture was treated with glucoamylase, an enzyme that hydrolyzes α-1,4-linkage between glucose and glucose, the peak area of products 2, 4 and 5 decreased (Fig. 3B). It suggested that products 2, 4 and 5 contained at least two glucose units covalently linked by α-1,4-glycosidic linkage. On the contrary, the peak area of products 1 and 3 increased, suggesting that they were not hydrolyzed by glucoamylase and should be fisetin monoglucosides. To further hydrolyze the remaining glucoside products, the reaction mixture was treated with α-glucosidase after the glucoamylase treatment. and no product peaks were observed (Fig. 3C). This result indicated that the first glucosyl residue covalently linked to fisetin *via* α-1,4-linkage.

**Figure 4:**
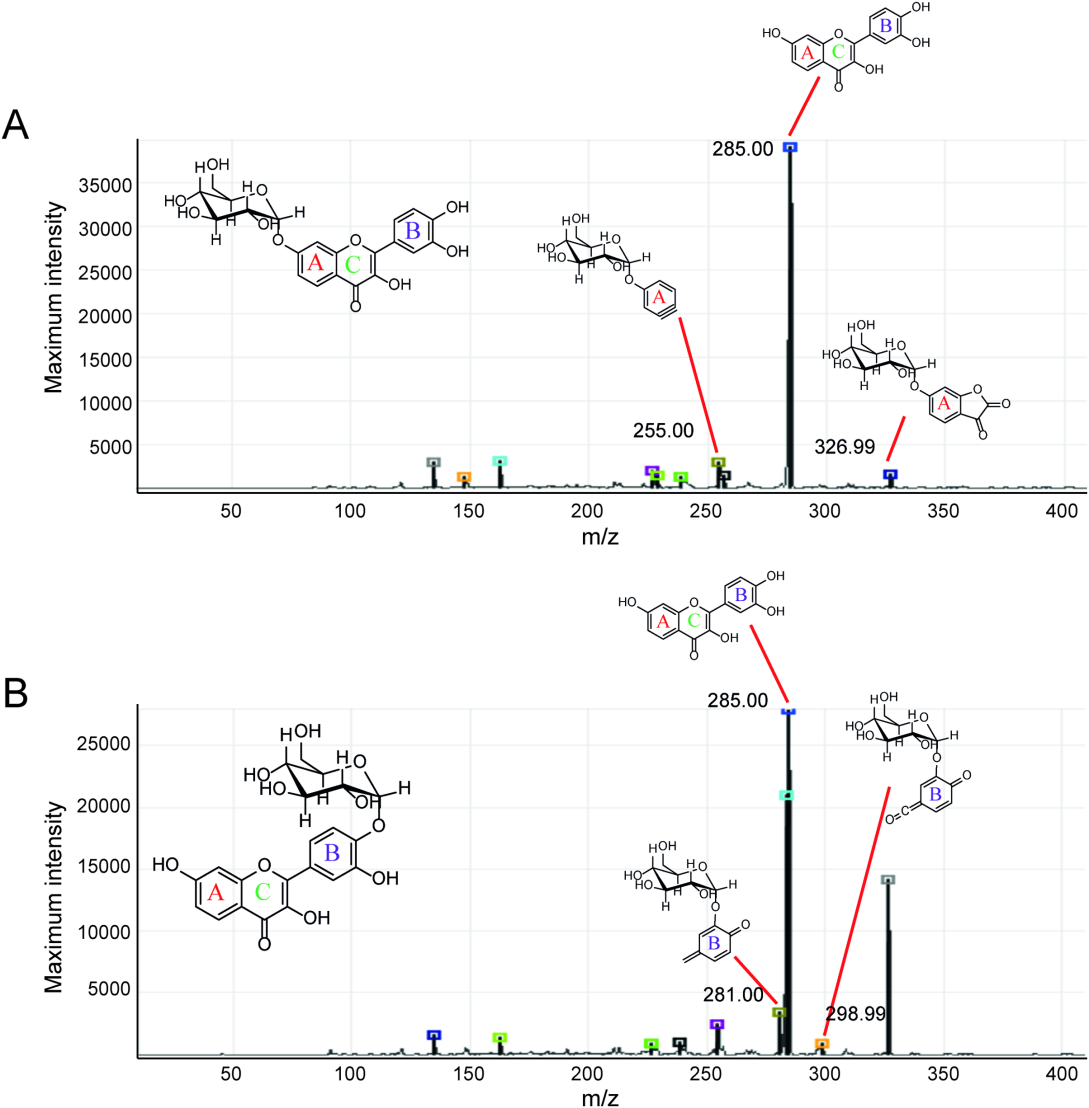
MS^2^ spectrum of parent ion at m/z 447.08. (A) Product 1 and (B) product 3.

Second, the isomer of obtained fisetin monoglycoside was then evaluated by fragmentation pattern using LC-MS/MS. MS^n^ has successfully been implemented to elucidate the structure of various flavonoids and their glycoside derivatives (Kachlicki et al., 2016). The fragmentation patterns of various flavonoids and their derivatives were studied, and the possible fragmentation patterns of fisetin were predicted (Fig. S8). Usually, only labile bonds undergo fragmentation during MS^2^ experiments. The purified fractions of products 1 and 3 were subjected to MS/MS analysis since they were less complicated among all products. As shown in Fig. 4, the structure of product 1 could be differentiated from product 3 since they showed different MS^2^ fragment patterns. Product 1 was creditable to be the fisetin-7-O-glucoside since the MS^2^ showed the ion at m/z 285, 255, 327 which corresponded to the mass of [fisetin], [^0,4^A + Glc] and [^1,2^A + Glc] ion, respectively (Fig. 4A, Fig. S8A). For product 3, it was suggested as fisetin-4’-O-glucoside because the fragment pattern of MS^2^ showed the ion at m/z 285, 281, 299 which correlated to the mass of [fisetin], [^0,2^B + Glc] and [^1,2^B + Glc], respectively (Fig. 4B, Fig. S8B). The MS^2^ spectrum of product 3 also showed the ion at m/z 328 which corresponded to the fragmentation of the glucosyl moiety of fisetin monoglucoside (Fig. S8C). In comparison to previous studies, it was found that fisetin glycosides synthesized from engineered *E. coli* were both fisetin 3-O-glucoside and fisetin 3-O-rhamnoside, indicating that the molecular structure of fisetin glycosides is largely dependent on the catalysts used (Pandey et al., 2016).

### Molecular docking study

**Figure 5:**
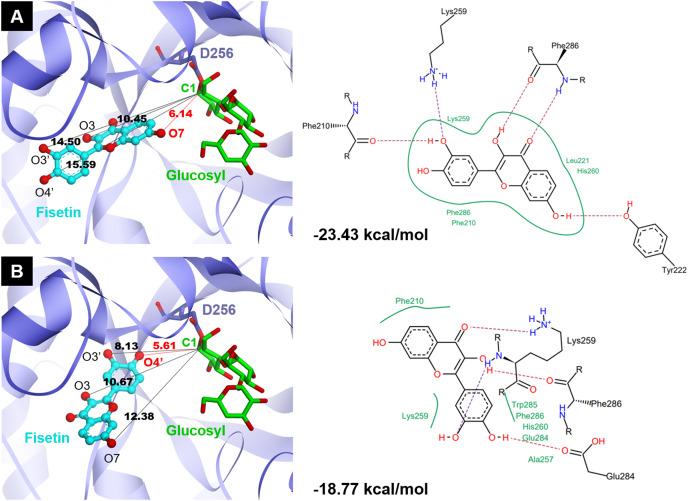
Two binding modes of fisetin found from top 20 ligand conformers. (A) The first binding mode: O7 atom of ring A turns toward C1 atom of the first glucosyl residue of glycosyl-D256 intermediate. (B) The second binding mode: O4′ atom of ring B turns toward C1 atom of the first glucosyl residue of glycosyl-D256 intermediate.

Molecular docking analysis was carried out in order to illustrate the binding conformation of fisetin in CGTase active site. The top 20 ligand conformers with the lowest docking energy were selected from 500 runs. The result showed that there were two ligand binding modes found from top 20 ligand conformers. The first binding mode of fisetin turns O7 atom of ring A of fisetin toward C1 atom of the first glucosyl residue of glycosyl-D256 intermediate (Fig. 5A). The distance between O1-C1 is 6.14 Å. For the second mode (Fig. 5B), O4’ atom of ring B turns toward C1 atoms of the first glucosyl residue of glycosyl-D256 intermediate, and the O4’-C1 distance is 5.16 Å. The result suggested that CGTase could transfer glycosyl moieties to O7 and O4’ atoms of fisetin, producing product 1 and product 3, respectively. These results corresponded to the structures of fisetin monoglucoside analyzed by MS/MS. Moreover, the docking energies demonstrated that the first binding mode was more stable than the second mode since it showed the lower docking energy (−23.43 kcal/mol and −18.77 kcal/mol). It indicated that transglycosylation of CGTase possibly preferred the O7 of ring A to O4’ of ring B, resulting in the higher amount of product 1 than product 3, which corresponded to the HPLC peak area (Fig. 3) (Ortiz-Soto et al., 2020; Rha et al., 2019; Rha et al., 2020).

### Water solubility and antioxidant activity of fisetin glycosides

After fisetin glycosides were synthesized and purified, the water solubility and antioxidant activity of glycoside products were then analyzed and compared to those of the original fisetin. Since the yield of fisetin di- and triglucoside is relatively low, the solubility and antioxidant activity were studied only in fisetin monoglucosides. The purified products 1 and 3 (fisetin monoglucosides) were dissolved in deionized water. The molar concentration of fisetin glycosides was determined from molar concentration of fisetin by HPLC after they were hydrolyzed by acid. The result showed that at least 830 and 410 µM of products 1 and 3 were able to dissolve in water while the original fisetin was able to dissolve at a concentration as low as 0.46 µM. It can evidently be seen that products 1 and 3 had water solubility of at least 1,800 and 888 times higher than that of fisetin.

After that, DPPH assay was used to determine the antioxidant activity of these fisetin monoglucosides. The results showed that the concentration of obtained fisetin monoglucosides (products 1 and 3) required for scavenging radicals of DPPH by 50% (IC50) was 2.28 and 2.52 µM, respectively, while the IC50 of fisetin was 2.72 µM (Table 1). This result showed that the antioxidant activity of parental fisetin and fisetin glycosides was comparable. Transglycosylation by CGTase displayed a minor effect on the scavenging activity of fisetin. In comparison to other studies, transglycosylation usually decreases the antioxidant activity of flavonoids. For example, epicatechin glucosides synthesized by CGTase showed 1.5-fold lower antioxidant activity than that of parental epicatechin (Aramsangtienchai et al., 2011). Resveratrol-3-O-glucoside and resveratrol-3-O-maltoside showed 1.1- and 1.9-fold lower antioxidant activity than that of the parent resveratrol (Sato et al., 2014). Moreover, the IC50 of fisetin and fisetin glucosides is relatively low compared to other flavonoids (Table S2). This result demonstrated that fisetin glucoside synthesized by CGTase has high antioxidant activity, which should be valuable for pharmaceutical and functional food industries.

**Table 1:** Antioxidant activity of fisetin and derived fisetin glycosides synthesized by CGTase from *Paenibacillus sp*. RB01.

Compound	Structure	IC_50_ (µM)
Fisetin	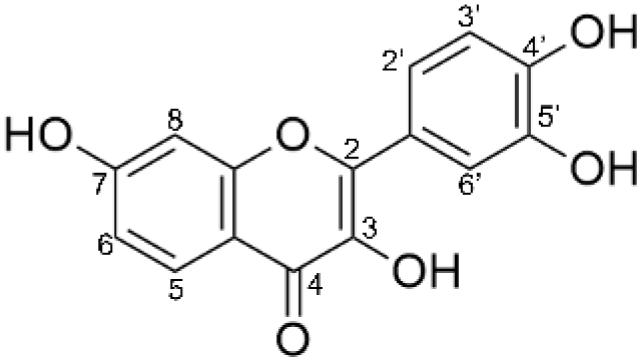	2.72 ± 0.21
Product 1 (fisetin-7-O-glucoside)	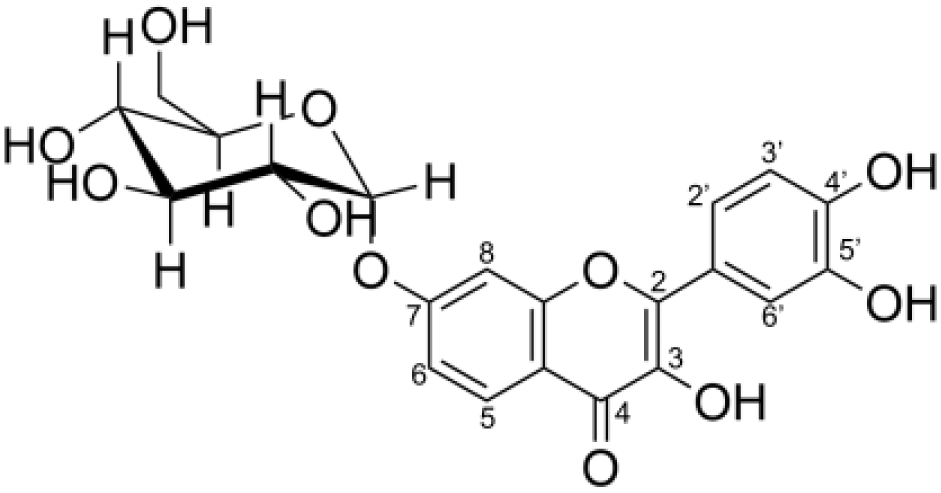	2.28 ± 0.72
Product 3 (fisetin-4′-O-glucoside)	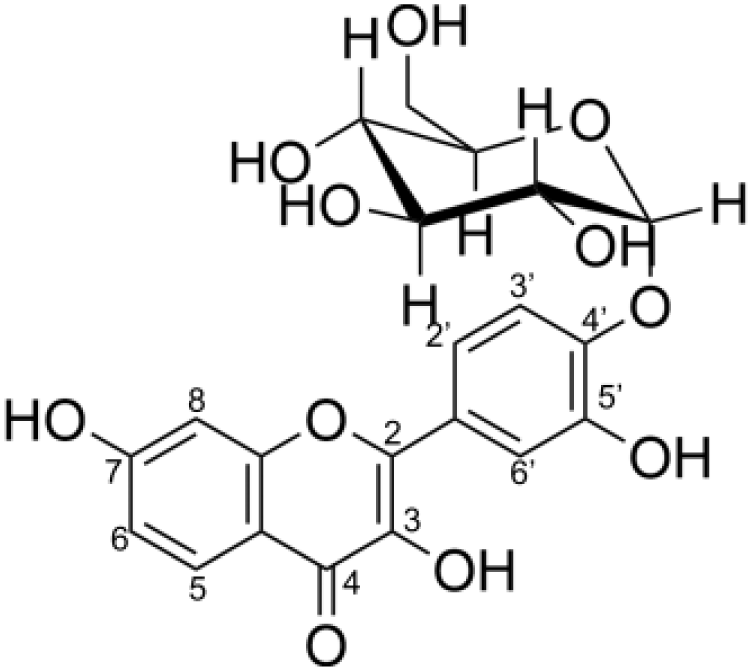	2.52 ± 0.48

## Conclusions

Our results demonstrated that CGTase from *Paenibacillus* sp. RB01 has the capability to synthesize fisetin glycosides *via* coupling reaction using β-CD as a glycosyl donor. The products were separated, purified, and characterized. At least five different glycoside derivatives were formed, and at least two species of fisetin monoglucosides were found to possess high water solubility and still express high antioxidant activity compared to that of fisetin. This finding revealed that the synthesis of fisetin glycosides by transglycosylation reaction of CGTase is very attractive and could be applied for commercial developments of other flavonoid glycosides.

## Supplemental Information

10.7717/peerj.13467/supp-1 Supplemental Information 1 Supplemental Figures and Tables.

10.7717/peerj.13467/supp-2 Supplemental Information 2 DNA and amino acid sequence of CGTase from *Paenibacillus* sp. RB01.

10.7717/peerj.13467/supp-3 Supplemental Information 3 Raw data. The relative activity of enzyme after incubating in different concentration of organic solvent, DMSO and acetone, in triplicate. The average value of the data was plotted in Fig. 1.

## Additional Information and Declarations

### Data Availability

The following information was supplied regarding data availability: The raw data is available in the Supplemental File.
