# Supplementary material for: Fisetin glycosides synthesized by cyclodextrin glycosyltransferase from Paenibacillus sp. RB01: characterization, molecular docking, and antioxidant activity
Source: PeerJ. 2022 May 24;10:e13467. doi: 10.7717/peerj.13467 (PMC9147316; doi:10.7717/peerj.13467)
Supplement: Supplemental Information 1 [file peerj-10-13467-s001.docx]

Supporting information

Fisetin glycosides synthesized by cyclodextrin glycosyltransferase from Paenibacillus sp. RB01: Characterization, molecular docking and antioxidant activity

Nattawadee Lorthongpanich^1^, Panupong Mahalapbutr^2^, Thanyada Rungrotmongkol^3^, Thanapon Charoenwongpaiboon^4^*, Manchumas Hengsakul Prousoontorn^1^*

^1^ Department of Biochemistry, Faculty of Science, Chulalongkorn University, Pathumwan, Bangkok,10330, Thailand

^2^ Department of Biochemistry, Center for Translational Medicine, Faculty of Medicine, Khon Kaen University, Khon Kaen 40002, Thailand

^3^ Structural and Computational Biology Research Unit, Department of Biochemistry, Faculty of Science, Chulalongkorn University, Bangkok 10330, Thailand

^4^ Department of Chemistry, Faculty of Science, Silpakorn University, Nakhon Pathom, 73000, Thailand


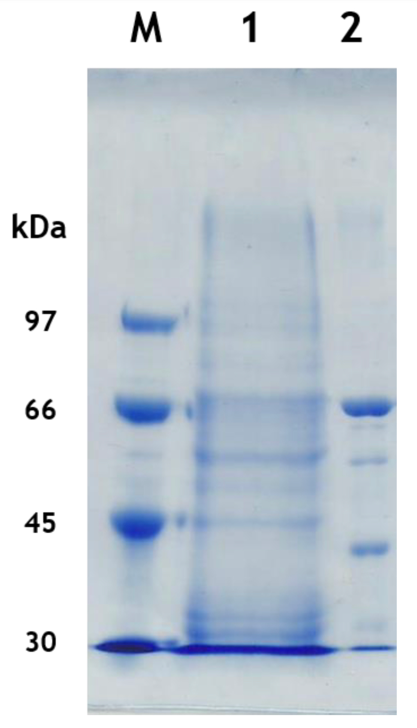


Figure S1. SDS-PAGE of purified CGTase: lane M molecular weight markers; lane 1 crude enzyme; lane 2 purified enzyme.

Table S1 Purification data for CGTase.

| Step | Total activity (U) | Total protein (mg) | Specific activity (U/mg) | Purification (fold) | Yield (%) |
| --- | --- | --- | --- | --- | --- |
| Crude | 80,300 | 549 | 146 | 1 | 100 |
| Starch  adsorption | 24,700 | 4.90 | 5030 | 34 | 31 |


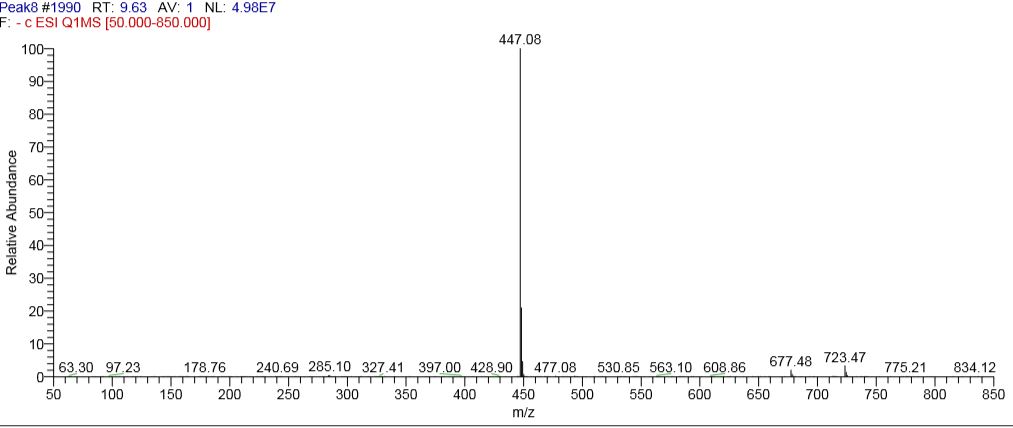


Figure S2. ESI/MS^1^ analysis profile in negative mode ionization of the product 1 (retention time of 16.5 minutes).


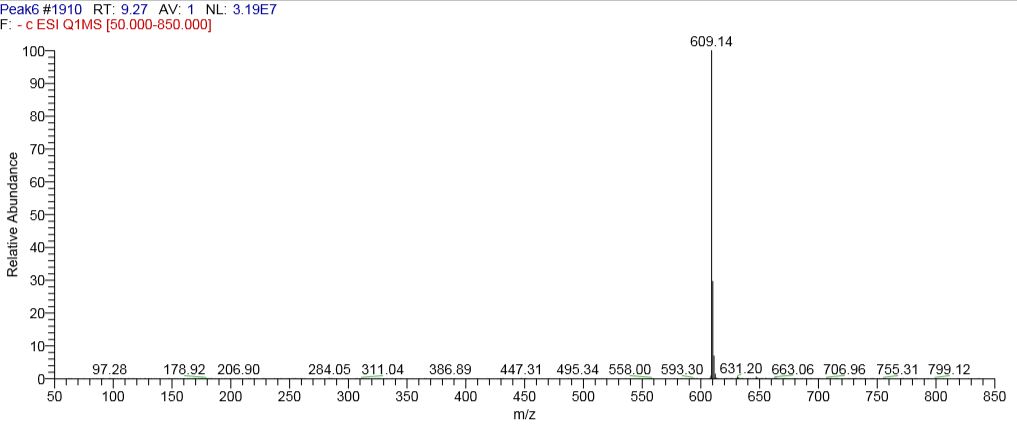


Figure S3. ESI/MS^1^ analysis profile in negative mode ionization of the product 2 (retention time of 15.3 minutes).


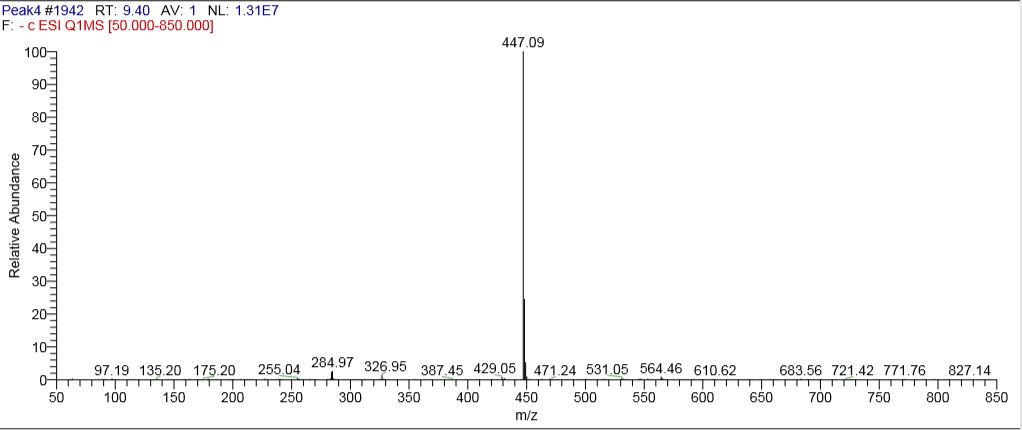


Figure S4. ESI/MS^1^ analysis profile in negative mode ionization of the product 3 (retention time of 14.7 minutes).


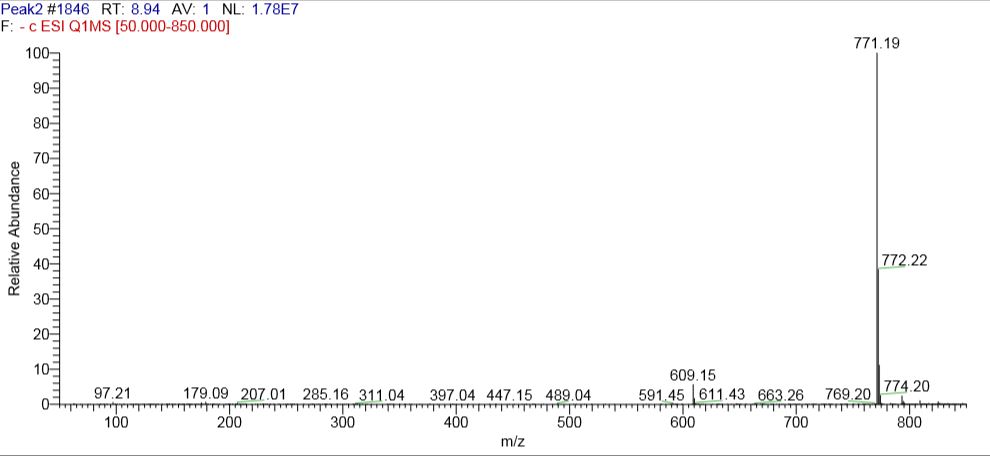


Figure S5. ESI/MS^1^ analysis profile in negative mode ionization of the product 4 (retention time of 13.9 minutes).


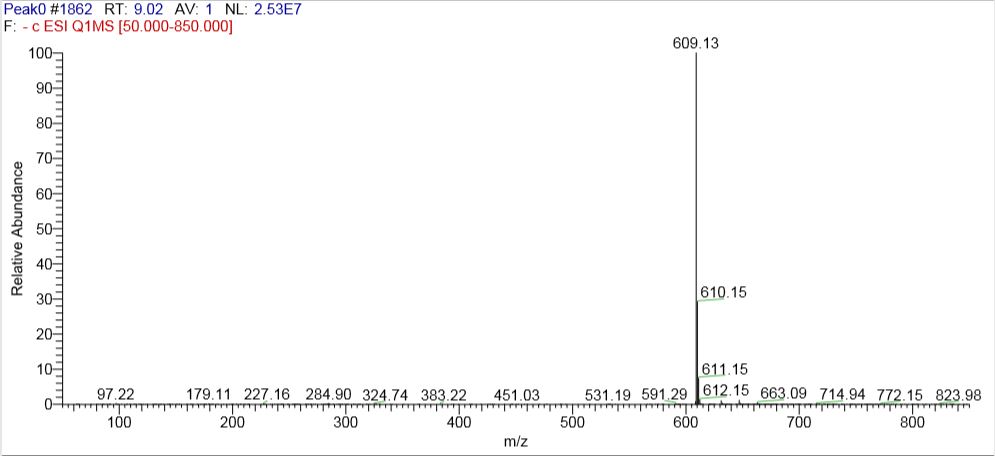


Figure S6. ESI/MS^1^ analysis profile in negative mode ionization of the product 5 (retention time of 13.4 minutes).


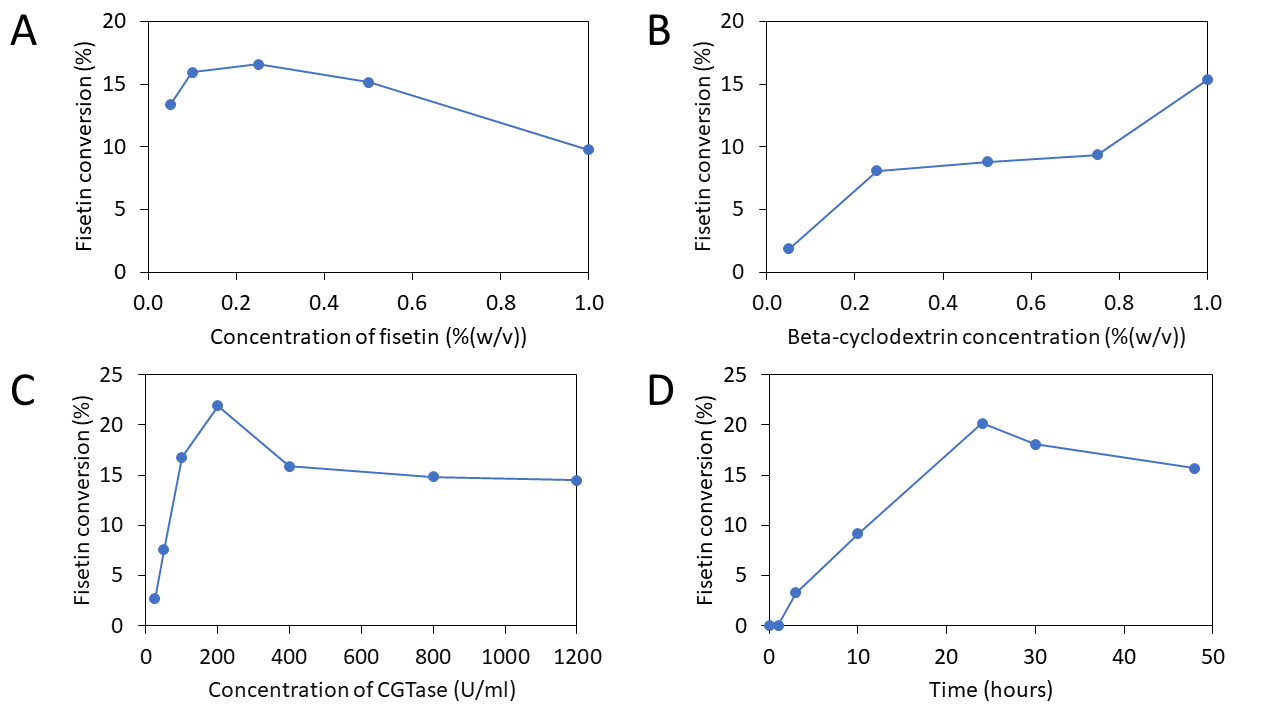


Figure S7. The effect of fisetin concentration (A), β-cyclodextrin concentration (B), CGTase concentration (C) and incubation time (D) on fisetin conversion (%). The remaining fisetin was determined by HPLC.

| 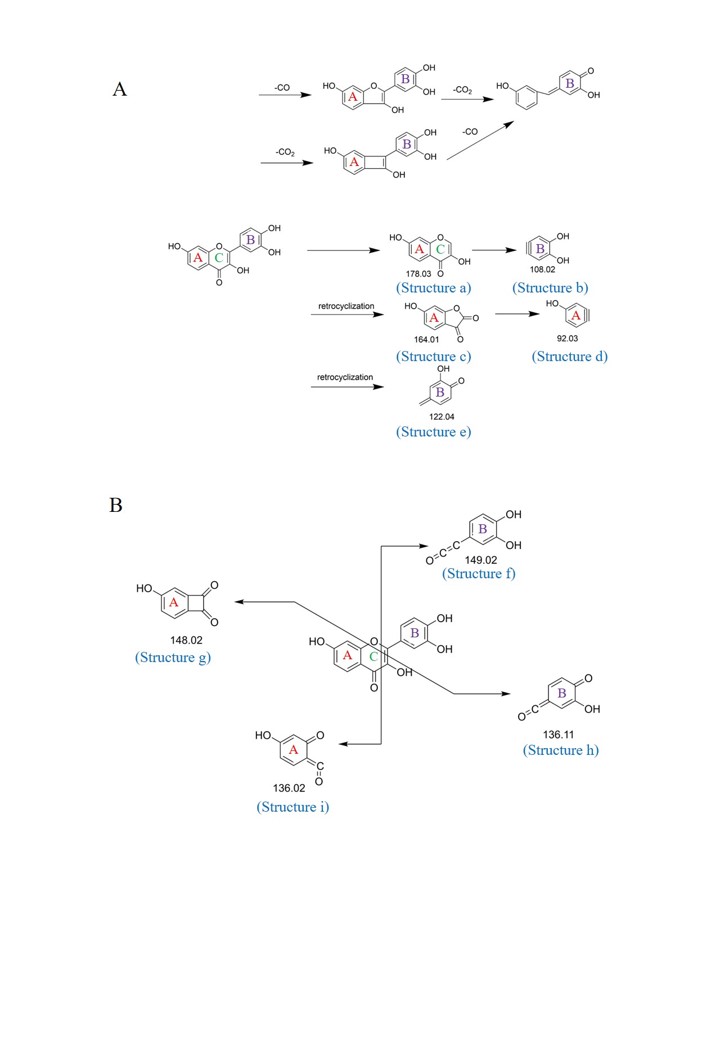 | 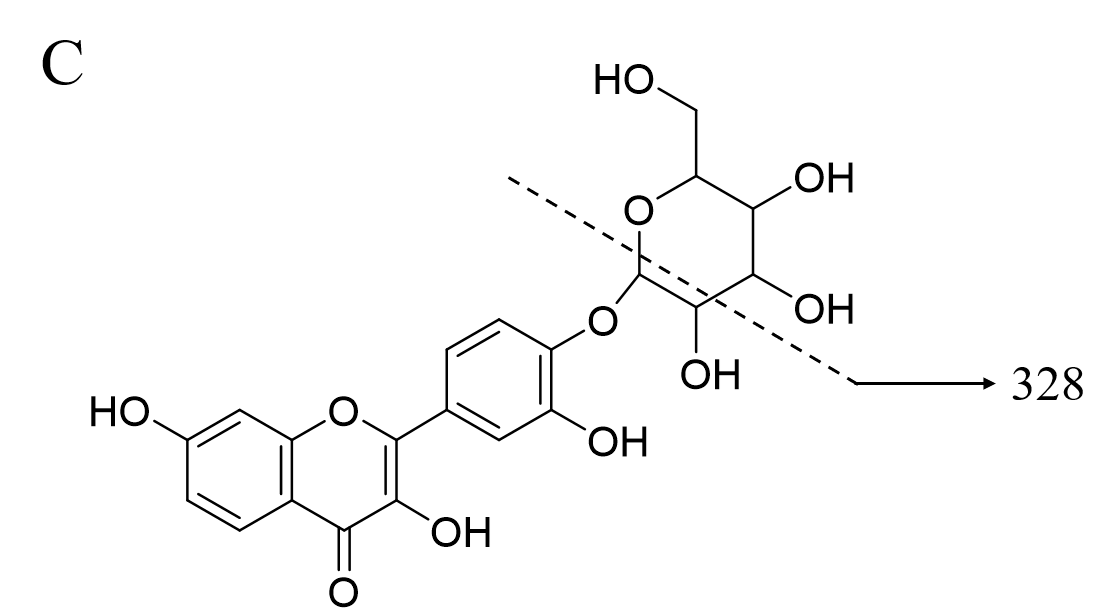 |
| --- | --- |

Figure S8. Prediction of fragmentation patterns of fisetin derived from mass spectroscopy. (A) The fragment modified from quercetin (Kometani et al. 1994), (B) Formal notation used for fragmentation of the [M − H]^−^ ions of flavonoids (Troalen et al. 2014), and (C) the predicted mass of ion that should be obtained from fragmentation of the first glucosyl moiety of fisetin monoglucoside.

Table S2 The IC50 value of DPPH radical scavenging activity of some flavonoid and their glycosides

| Compound | IC_50_ for DPPH (µM) | References |
| --- | --- | --- |
| Fisetin | 2.72 | This work |
| Fisetin glucosides | 2.3 and 2.5 | This work |
| Epicatechin | 76.5 | (Aramsangtienchai et al. 2011) |
| Epicatechin glucosides | 115 | (Aramsangtienchai et al. 2011) |
| Resveratrol | 57.8 | (Iacopini et al. 2008) |
| Catechin | 6.7 | (Iacopini et al. 2008) |
| Rutin | 7.4 | (Iacopini et al. 2008) |
| Quercetin | 5.5 | (Iacopini et al. 2008) |

**References**

Aramsangtienchai P, Chavasiri W, Ito K, and Pongsawasdi P. 2011. Synthesis of epicatechin glucosides by a β-cyclodextrin glycosyltransferase. *Journal of Molecular Catalysis B: Enzymatic* 73:27-34. <https://doi.org/10.1016/j.molcatb.2011.07.013>

Iacopini P, Baldi M, Storchi P, and Sebastiani L. 2008. Catechin, epicatechin, quercetin, rutin and resveratrol in red grape: Content, in vitro antioxidant activity and interactions. *Journal of Food Composition and Analysis* 21:589-598. <https://doi.org/10.1016/j.jfca.2008.03.011>

Kometani T, Terada Y, Nishimura T, Takii H, and Okada S. 1994. Transglycosylation to hesperidin by cyclodextrin glucanotransferase from an alkalophilic bacillus species in alkaline pH and properties of hesperidin glycosides. *Bioscience, Biotechnology, and Biochemistry* 58:1990-1994. 10.1271/bbb.58.1990

Troalen LG, Phillips AS, Peggie DA, Barran PE, and Hulme AN. 2014. Historical textile dyeing with Genista tinctoria L.: a comprehensive study by UPLC-MS/MS analysis. *Analytical Methods* 6:8915-8923. 10.1039/C4AY01509F
