# Supplementary material for: Fisetin glycosides synthesized by cyclodextrin glycosyltransferase from Paenibacillus sp. RB01: characterization, molecular docking, and antioxidant activity
Source: PeerJ. 2022 May 24;10:e13467. doi: 10.7717/peerj.13467 (PMC9147316; doi:10.7717/peerj.13467)
Supplement: Supplemental Information 2 [file peerj-10-13467-s002.docx]

atgaaaagatttatgaaactaacagccgtatggacactctggttatccctcacgctgggc 60

M K R F M K L T A V W T L W L S L T L G 20

ctcttgagcccggtccacgcagccccggatacctcggtatccaacaagcagaatttcagc 120

L L S P V H A A P D T S V S N K Q N F S 40

acggatgtcatatatcagatcttcaccgaccggttctcggacggcaatccggccaacaat 180

T D V I Y Q I F T D R F S D G N P A N N 60

ccgaccggcgcggcatttgacggatcatgtacgaatcttcgcttatactgcggcggcgac 240

P T G A A F D G S C T N L R L Y C G G D 80

tggcaaggcatcatcaacaaaatcaacgacggttatttgaccggcatgggcattacggcc 300

W Q G I I N K I N D G Y L T G M G I T A 100

atctggatttcacagcctgtcgagaatatctacagcgtgatcaactactccggcgtccat 360

I W I S Q P V E N I Y S V I N Y S G V H 120

aatacggcttatcacggctactgggcgcgggacttcaagaagaccaatccggcctacgga 420

N T A Y H G Y W A R D F K K T N P A Y G 140

acgatgcaggacttcaaaaacctgatcgacaccgcgcatgcgcataacataaaagtcatc 480

T M Q D F K N L I D T A H A H N I K V I 160

Atcgactttgcaccgaaccatacatctccggcttcttcggatgatccttcctttgcagag 540

I D F A P N H T S P A S S D D P S F A E 180

Aacggccgcttgtacgataacggcaacctgctcggcggatacaccaacgatacccaaaat 600

N G R L Y D N G N L L G G Y T N D T Q N 200

Ctgttccaccattatggcggcacggatttctccaccattgagaacggcatttataaaaac 660

L F H H Y G G T D F S T I E N G I Y K N 220

Ctgtacgatctggctgacctgaatcataacaacagcagcgtcgatgtgtatctgaaggat 720

L Y D L A D L N H N N S S V D V Y L K D 240

gccatcaaaatgtggctcgacctcggggttgacggcattcgcgtggacgcggtcaagcat 780

A I K M W L D L G V D G I R V D A V K H 260

atgccattcggctggcagaagagctttatgtccaccattaacaactacaagccggtcttc 840

M P F G W Q K S F M S T I N N Y K P V F 280

accttcggcgaatggttccttggcgtcaatgagattagtccggaataccatcaattcgct 900

T F G E W F L G V N E I S P E Y H Q F A 300

aacgagtccgggatgagcctgctcgatttccgctttgcccagaaggcccggcaagtgttc 960

N E S G M S L L D F R F A Q K A R Q V F 320

agggacaacaccgacaatatgtacggcctgaaagcgatgctggagggctctgaagtagac 1020

R D N T D N M Y G L K A M L E G S E V D 340

tatgcccaggtgaatgaccaggtgaccttcatcgacaatcatgacatggagcgtttccac 1080

Y A Q V N D Q V T F I D N H D M E R F H 360

accagcaatggcgacagacggaagctggagcaggcgctggcctttaccctgacttcacgc 1140

T S N G D R R K L E Q A L A F T L T S R 380

ggtgtgcctgccatctattacggcagcgagcagtatatgtctggcgggaatgatccggac 1200

G V P A I Y Y G S E Q Y M S G G N D P D 400

aaccgtgctcggattccttccttctccacgacgacgaccgcatatcaagtcatccaaaag 1260

N R A R I P S F S T T T T A Y Q V I Q K 420

ctcgctccgctccgcaaatccaacccggccatcgcttacggttccacacaggagcgctgg 1320

L A P L R K S N P A I A Y G S T Q E R W 440

atcaacaacgatgtgatcatctatgaacgcaaattcggcaataacgtggccgttgttgcc 1380

I N N D V I I Y E R K F G N N V A V V A 460

attaaccgcaatatgaacacaccggcttcgattaccggccttgtcacttccctcccgcag 1440

I N R N M N T P A S I T G L V T S L P Q 480

ggcagctataacgatgtgctcggcggaattctgaacggcaatacgctaaccgtgggtgct 1500

G S Y N D V L G G I L N G N T L T V G A 500

ggcggtgcagcttccaactttactttggctcctggcggcactgctgtatggcagtacaca 1560

G G A A S N F T L A P G G T A V W Q Y T 520

accgatgccacagctccgatcatcggcaatgtcggcccgatgatggccaagccaggggtc 1620

T D A T A P I I G N V G P M M A K P G V 540

Acgattacgattgacggccgcggcttcggctccggcaagggaacggtttacttcggtaca 1680

T I T I D G R G F G S G K G T V Y F G T 560

acggcagtcactggcgcggacatcgtagcttgggaagatacacaaatccaggtgaaaatc 1740

T A V T G A D I V A W E D T Q I Q V K I 580

cctgcggtccctggcggcatctatgatatcagagttgccaacgcagccggagcagccagc 1800

P A V P G G I Y D I R V A N A A G A A S 600

aacatctacgacaatttcgaggtgctgaccggagaccaggtcaccgttcggttcgtaatc 1860

N I Y D N F E V L T G D Q V T V R F V I 620

aacaatgccacaacggcgctgggacagaatgtgttcctcacgggcaatgtcagcgagctg 1920

N N A T T A L G Q N V F L T G N V S E L 640

ggcaactgggatccgaacaacgcgatcggcccgatgtataatcaggtcgtctaccaatac 1980

G N W D P N N A I G P M Y N Q V V Y Q Y 660

ccgacttggtattatgatgtcagcgttccggcaggccaaacgattgaatttaaattcctg 2040

P T W Y Y D V S V P A G Q T I E F K F L 680

aaaaagcaaggctccaccgtcacatgggaaggcggcgcgaatcgcaccttcaccacccca 2100

K K Q G S T V T W E G G A N R T F T T P 700

accagcggcacggcaacgatgaatgtgaactggcagccttaa 2142

T S G T A T M N V N W Q P - 713
